# Mutational landscape of the G-quadruplex DNA in *cMyc* proto-oncogene promoter: Insights into the structural polymorphism and transcriptional regulation

**DOI:** 10.1016/j.jbc.2025.110897

**Published:** 2025-11-04

**Authors:** Ai-Min Su, Qiu-Ying Chen, Xue-Yang Yu, Wen-Jing Lu, Dong Wang, Xi-Miao Hou

**Affiliations:** College of Life Sciences, Northwest A&F University, Yangling, China

**Keywords:** G-quadruplex, single-nucleotide variation, *cMyc* promoter, gene regulation, polymorphism

## Abstract

Noncanonical four-stranded DNA structures known as G-quadruplexes (G4s) play crucial roles in gene regulation and have recently been identified as hotspots for single-nucleotide variations (SNVs). In the *cMyc* proto-oncogene promoter, the Pu27 G4 element serves as a key transcriptional switch, yet its susceptibility to SNVs and the resulting molecular consequences remain unclear. Here, we systematically analyze SNVs in the *cMyc* promoter G4 using the dbSNP database and identify 17 mutation sites distributed across its 27-nt sequence, indicating a high mutation frequency. Biophysical studies reveal that SNVs in loop regions primarily enhance G4 stability by introducing additional hydrogen bonds and π-π interactions while maintaining its parallel topology. In contrast, SNVs within G-runs destabilize the G4, leading to structural polymorphism, mixed topologies, and increased conformational dynamics; certain SNVs induce noncanonical G4 architectures such as G-vacancies and bulges. Functional assays show that these structural changes differentially modulate transcription from the *cMyc* promoter, with stabilizing SNVs generally suppressing expression and destabilizing SNVs producing divergent effects. Overall, our findings establish G4s as dynamic sensors of genetic variation and provide a mechanistic framework for understanding how non-B DNA motifs contribute to transcriptional dysregulation and genome instability in cancer.

G-quadruplexes (G4s) are noncanonical four-stranded nucleic acid structures formed by guanine-rich sequences through Hoogsteen hydrogen bonding. They are widely distributed throughout the genome, particularly in promoters, telomeres, and regulatory regions (1), where they play vital roles in regulating transcription, replication, and maintaining genome stability (2). High-throughput sequencing studies have identified more than 700,000 putative G4-forming sequences in the human genome, highlighting their widespread presence and functional significance (3). The stability and formation of G4s are influenced by sequence composition, ionic conditions, and interactions with proteins and small molecules, making them dynamic regulators of gene expression (4).

Single-nucleotide variations (SNVs) are among the most common genetic mutations and play a critical role in human diseases, including cancer and genetic disorders (5, 6). Growing evidence suggests that G4 structures are hotspots for SNVs, with significant implications for gene regulation and genome stability (7, 8, 9, 10, 11). For instance, Du *et al.* identified G4s as mutational hotspots associated with increased mutation rates and transcriptional variability (11), while Guiblet *et al.* demonstrated that sequence variations in non-B DNA motifs, particularly G4s, shape both local and global mutation landscapes (12). Moreover, Bacolla *et al.* linked G4-forming sequences to chromosomal breakpoints in cancer genomes, highlighting their relevance in genomic instability and tumorigenesis (13). Beyond their prevalence, SNVs within G4s have been shown to directly influence gene expression and structural dynamics. Gong *et al.* reported that SNV-induced alterations in G4 folding impact transcriptional regulation (14), while Baral *et al.* demonstrated that such variations contribute to inter-individual differences in gene expression (15). Additionally, Zhang *et al.* revealed that G4 structures modulate somatic structural variants, reinforcing their role in genome instability (16). These findings underscore the regulatory potential of SNV-G4 interactions, yet their molecular consequences remain underexplored, particularly in oncogenes where G4s play critical regulatory roles.

The *cMyc* proto-oncogene encodes a transcription factor essential for cell proliferation, differentiation, and apoptosis. Its promoter contains a well-characterized G4-forming sequence, Pu27 (NHE III1), which acts as a transcriptional switch by adopting a stable G4 conformation that represses *cMyc* expression (17, 18). Stabilization of this structure by small molecules has been proposed as an anticancer strategy to suppress *cMyc* overexpression in tumors (19, 20). However, the regulatory role of this G4 is now understood to be more complex. A recent study using CRISPR-based disruption at the endogenous locus revealed that the *cMyc* promoter G4 can also act as a positive regulator, potentially by recruiting transcription factors and chromatin-modifying enzymes to activate transcription (21). Despite extensive studies of the *cMyc* promoter G4, its role as a mutation hotspot and the consequences of naturally occurring SNVs within this element are still poorly understood. Given its critical involvement in oncogenesis and its promise as a therapeutic target, it is essential to understand how SNVs modify the structural and regulatory properties of the *cMyc* promoter G4. Key questions include how these variations influence G4 stability, folding architecture, and dynamics as well as their downstream effects on transcriptional regulation and genome integrity.

In our study, we conducted a systematic investigation into the structural and functional consequences of SNVs within the *cMyc* promoter G4 (Table S1). By analyzing the dbSNP database, we identified multiple naturally occurring SNVs within the G4-forming sequence and investigated their impact using a combination of biophysical (CD spectroscopy, NMR, smFRET, and DMS-footprinting) and functional assays (*Gaussia* luciferase reporter and polymerase stop assays). Our analyses revealed dual effects of SNVs: variations in loop regions enhance G4 thermostability through additional hydrogen bonding and π–π interactions, while alterations in the G-run segments destabilize the structure, leading to increased conformational heterogeneity and mixed topologies. Functionally, SNVs within the *cMyc* promoter G4 can substantially modulate transcription. Stabilizing SNVs that maintain the overall topology tend to suppress gene expression, while destabilizing SNVs produce heterogeneous effects, sometimes relieving repression, but in certain contexts further reducing transcription. Collectively, these results underscore the dynamic nature of G4 structures as sensitive sensors of genetic variation and highlight their potential as targets in *cMyc*-driven cancers.

## Results

### Mutational landscape of G4 DNA in *cMyc* proto-oncogene promoter

**Figure 1:**
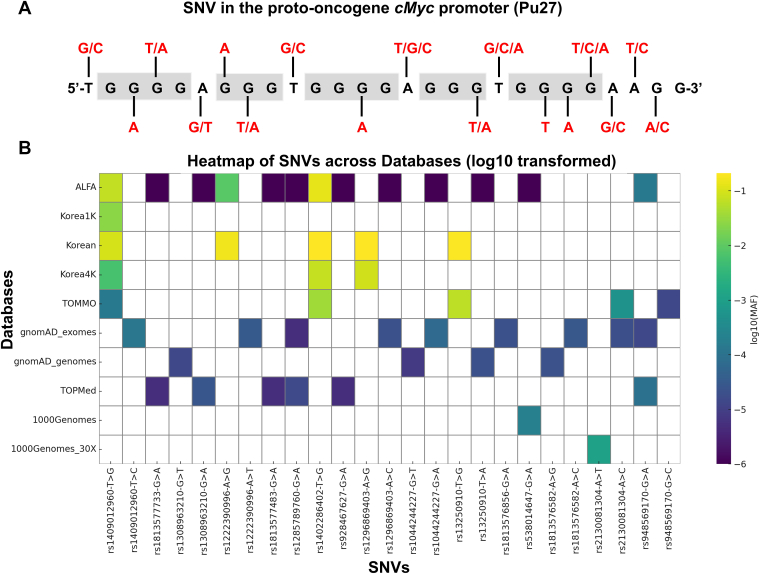
**SNVs in the *cMyc* promoter G4.***A*, schematic of naturally occurring single-nucleotide variations (SNVs) within the Pu27 G4-forming element (NHE III1) of the *cMyc* promoter, as identified in the dbSNP database. The reference G-tracts are shown in *gray boxes*, with the alternative alleles highlighted in *red*. *B*, heatmap showing the minor allele frequencies (log10-transformed) of these SNVs across multiple population databases, including ALFA, Korea1K, Korean, Korea4K, TOMMO, gnomAD (exomes and genomes), TOPMed, and 1000 Genomes. *White* color indicates the data are unavailable in the corresponding database.

To systematically investigate the distribution of SNVs in the *cMyc* promoter G4 region, we analyzed publicly available SNV datasets from the dbSNP database, covering both SNVs and small insertions/deletions (indels). This 27-nucleotide sequence emerged as a mutational hotspot with considerable variability (Table S2–S3). Minor allele frequencies (MAFs)—the proportion of chromosomes in a population carrying the less common allele at a given locus—were retrieved from the NCBI SNP database. While several indels with MAFs up to ∼0.66% were mapped (Fig. S1 and Table S3), SNVs were far more abundant, with 17 distinct mutation sites identified (Fig. 1*A*). These SNVs were distributed across both G-runs and loop regions. Notably, SNVs were detected at every base position within the loops, indicating full variant coverage of loop bases. Among the seven non-G loop bases, six carried variants involving substitution to guanine, a change likely to enhance G4 stability (Fig. 1*A*). This trend is further reflected in MAF values (Fig. 1*B*, Table S2); for example, rs1402286402 (T-to-G) and rs1296869403 (A-to-G) reach MAFs of 20.6% and 21.2% in Koreans, respectively, substantially higher than other loop substitutions.

Compared with loop regions, mutations within the G-runs were less frequent, generally showing MAFs below 0.02% (Fig. 1*B*, Table S2), indicative of strong purifying selection. Although rare in the general population, these variants occur within a structurally critical region, and their distinctive mutational patterns imply significant biological and medical relevance. Among these G-run mutations, the substitution spectrum in Figure 1*A* revealed a characteristic bias: Of all mutation types affecting the 10 mutable guanine residues, 60% were G-to-A transitions, followed by G-to-T (33%) and G-to-C (7%) transversions. This bias is also reflected in MAF data; for example, rs538014647 (G-to-A) and rs948569170 (G-to-A) have the highest frequencies in this category, at 0.02% and 0.0142%, respectively. This predominance of G-to-A substitutions highlights a mutational signature specific to this G4-forming sequence.

Collectively, these results demonstrate that Pu27 is a highly variable sequence, with SNVs enriched and exhibiting distinct positional and substitutional patterns. The low MAF observed in the G-run region reflects its strong evolutionary conservation and negative selective pressure, consistent with its essential role in maintaining G4 structural integrity. Mutations in this region are likely to have severe functional consequences. Conversely, the higher MAF in the loop region indicates greater tolerance for variation, which may facilitate regulatory diversity and adaptive flexibility.

Given the pronounced enrichment of SNVs in the *cMyc* promoter G4, it is crucial to determine how these variations alter G4 structure and function. To address this, we employed a truncated *cMyc* promoter G4 sequence (designated cMyc-G4), which adopts a well-characterized parallel-stranded quadruplex structure, as confirmed by PDB data (1XAV) and previous studies (22, 23, 24). This truncated sequence allows for a focused analysis of SNV effects while minimizing interference from flanking regions. Building on dbSNP analysis of natural variants, we further generated a systematic mutational landscape by substituting each nucleotide with the other three bases. The structural and functional consequences of these SNVs were examined using a combination of biophysical techniques, including circular dichroism (CD), nuclear magnetic resonance (NMR), dimethyl sulfate (DMS) footprinting, and single-molecule Förster resonance energy transfer (smFRET).

### Loop SNVs can enhance cMyc-G4 stability

**Figure 2:**
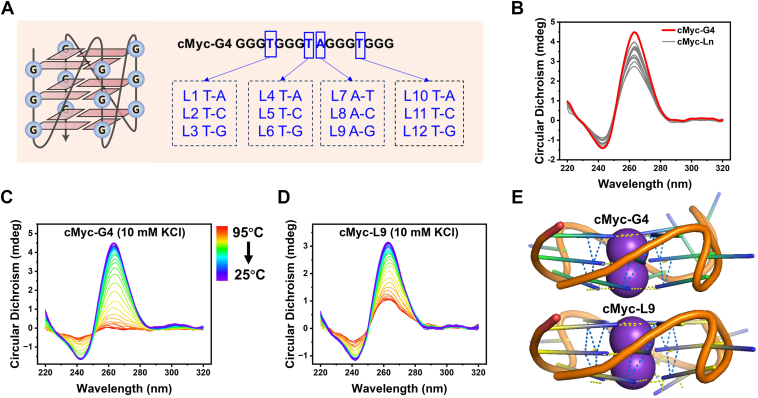
**Effect of loop SNV on G4 topology and thermostability.***A*, mutation sites in cMyc-G4. *B*, CD spectra of cMyc-G4 mutants (*gray*) showing similar topology to the wild type (*red*), with a positive peak at 263 nm and a negative valley near 240 nm. Measurements were performed with 4 μM DNA. *C* and *D*, melting curves of cMyc-G4 and the L9 mutant in 10 mM KCl. *E*, predicted structures of cMyc-G4 and the L9 mutant by AlphaFold 3 (pLDDT > 90). *Yellow dashed lines* indicate H-bonds; *blue dashed lines* indicate π-π stacking. Additional H-bonds and π-π stacking were observed in the L9 mutant.

To investigate the effects of SNVs in G4 loops, we first analyzed their impact on overall G4 topology (Fig. 2*A*). As expected, all loop mutants retained a G4 conformation similar to the wild type, as shown by CD spectra (Fig. 2*B*). This is because loop mutations do not disrupt the essential G-tetrads required for G4 formation. We next assessed the impact of loop SNVs on G4 thermostability using CD-melting assays. Since some mutants exhibited minimal changes in CD profiles at 100 mM KCl (data not shown), we conducted melting experiments in 10 mM KCl for better resolution (Fig. 2*C*). The wild-type G4 fully unfolded at 95 °C (Fig. S2*A*), while loop mutants displayed varying thermostability (Fig. S2, *B* and *C*). Mutants such as L2, L4, L5, L7, and L11 had melting behaviors similar to the wild type, with comparable *Tm* values. However, replacing non-G loop bases with guanine significantly enhanced thermostability (Fig. S2*C*). Notably, L3 and L12 resisted unfolding even at temperatures exceeding 90 °C, indicating a substantial stabilizing effect.

Native PAGE analysis confirmed that cMyc-G4 primarily exists as a monomer, with faster migration compared to intermolecular dimers (Fig. S3). A randomly selected loop mutant also formed an intramolecular G4 structure, as shown by gel filtration, consistent with the wild type (Fig. S3).

To further explore the structural basis of this enhanced stability, we employed AlphaFold 3 to predict the folding of loop mutants. As shown in Figure S4, AlphaFold 3 accurately predicted the G4 structures of both cMyc and human telomeric sequences, with high-confidence pLDDT scores exceeding 90, demonstrating the reliability of this method. Structural predictions of 12 loop mutants revealed that their G-runs and loop arrangements remained largely unchanged from the wild type (Fig. S5), even in sequences such as GGGTGGGTGGGGTGGG or GGGTGGGGAGGGTGGG. However, further analysis suggested that additional hydrogen bonding and π-π stacking interactions contributed to their increased stability (Table S4), consistent with previous reports (25, 26). For example, L9 exhibited enhanced stability due to these stabilizing interactions, as observed in Figures 2, *C*–*E* and Figure S5. In summary, while SNVs in loop regions do not alter the overall folding of cMyc-G4, they can significantly enhance thermostability by introducing stabilizing interactions, particularly when non-G loop bases are replaced with guanine.

### Tolerance of cMyc-G4 to SNVs in G-runs

**Figure 3:**
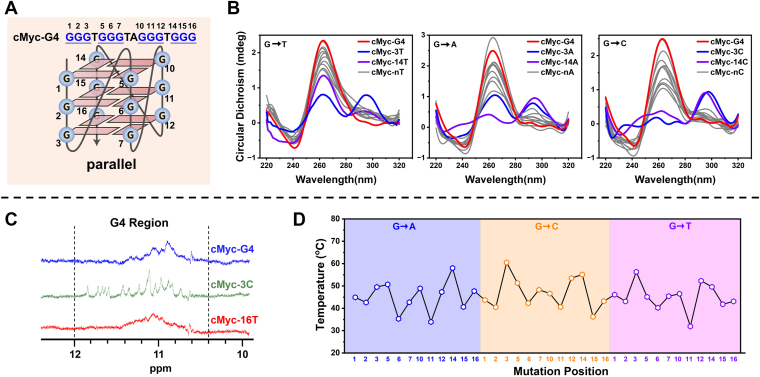
**Effect of G-run SNVs on G4 topology and thermostability.***A*, primary sequence and structural model of cMyc-G4, with mutation sites labeled. *B*, CD spectra of 4 μM mutants in 100 mM KCl at 25 °C. Most mutants (*gray*) retained a parallel conformation similar to the wild type (*red*), while some showed conformational changes (colored). nT, nA, and nC refer to other mutations. *C*, 1D ^1^H NMR spectra of cMyc-G4, 3C, and 16T (300 μM) in 100 mM KCl at 25 °C. Chemical shifts at 10.5 to 12.0 ppm confirm the Hoogsteen H-bond formation. *D*, *Tm* values of all G-run mutants (4 μM) determined by CD melting in 50 mM KCl. Wild-type cMyc-G4 had a *Tm* of 88.7 °C under these conditions.

To evaluate the structural impact of SNVs in the G-runs of cMyc-G4, we performed CD spectroscopy, G4-specific fluorescence probe binding assays, and ^1^H-NMR analysis. The CD spectra confirmed that all G-run mutants retained a G-quadruplex conformation (Fig. 3, *A* and *B*), a finding corroborated by the fluorescence probe assay (Fig. S6). Further validation using ^1^H-NMR spectroscopy indicated characteristic Hoogsteen hydrogen bonding signals (10.5–12.0 ppm, Fig. 3*C*) (27), demonstrating that despite sequence alterations, G4 folding was maintained. Although most SNVs preserved the parallel-stranded topology, subtle conformational changes were observed (Fig. 3*B*). Native PAGE analysis revealed that while all mutants primarily formed intramolecular G4 structures, some displayed slightly slower migration patterns, suggesting alterations in compactness (Fig. S7*A*). Additionally, gel filtration chromatography showed a leftward shift in elution volume for select mutants, indicating a looser structural organization than the wild-type G4 (Fig. S7, *B* and *C*).

Compared to the highly stable wild-type cMyc-G4 (*Tm* = 88.7 °C in 50 mM KCl), all SNV mutants showed a substantial reduction in melting temperature (Δ*Tm* > 25 °C, Fig. 3*D*), indicating a strong destabilizing effect. Notably, the impact of SNVs on G4 stability depended on their position within the G-runs, which could be categorized into three groups. First, SNVs near loop regions: mutations at G residues flanking the 1-nt loop (*e.g.*, 3T, 14T, 3A, 14A, 3C, 14C) expanded the loop to 2-nt, promoting the formation of non-parallel G4 conformations, as evidenced by changes in CD spectra (Fig. 3*A*). Second, SNVs at central G positions: mutations in the middle Gs of each G-run (positions 2, 6, 11, and 15) had the most severe destabilizing effects, causing a sharp drop in *Tm* (Fig. 3*D*). To investigate the mechanism, we performed DMS-footprinting on mutants 1T, 2T, and 3T (Fig. S8). In mutant 2T, both the fifth G and 14th G were highly cleaved (red arrows), indicating their exclusion from Hoogsteen hydrogen bonding. In contrast, only the fifth G was excluded in mutants 1T and 3T, resulting in their relatively higher *Tm*. Third, SNVs at other G sites: while mutations at other G positions had minimal effects on G4 folding, they still led to significant reductions in thermostability, highlighting the general destabilizing impact of SNVs in G-runs.

Overall, cMyc-G4 exhibits varying levels of tolerance to SNVs in its G-runs. Depending on the mutation site, structural reorganization can occur, leading to conformational switches and reduced stability.

### SNVs in G-runs enhance the structural dynamics and promote frequent state transitions

**Figure 4:**
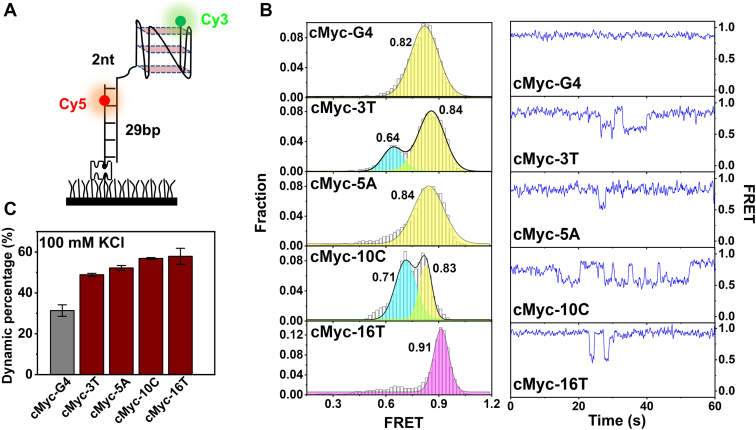
**Effects of G-run SNVs on G4 structural dynamics at the single-molecule level.***A*, schematic of smFRET substrates labeled with Cy3 and Cy5. *B*, FRET histograms (*left*) and representative FRET traces (*right*) of cMyc-G4 and mutants 3T, 5A, 10C, and 16T in 25 mM Tris-HCl buffer with 100 mM KCl at 22 °C. FRET histograms were fitted with single- or multi-peak Gaussian distributions; each histogram included at least 300 traces. *C*, Proportion of dynamic traces in 100 mM KCl. Data are shown as mean ± SD from three independent experiments.

Given the structural variations observed in G-run mutants, we next investigated their dynamic behavior using single-molecule Förster resonance energy transfer (smFRET) (28). This technique enabled real-time monitoring of conformational state transitions by strategically placing Cy3 and Cy5 fluorophores on the G4 structure (Fig. 4*A*). As expected, the wild-type cMyc-G4 exhibited a single, high-FRET efficiency peak (∼0.82), with only ∼30% of molecules displaying dynamic transitions within 1 min (Fig. 4, *B* and *C*). In contrast, SNV-containing mutants displayed broadened FRET distributions, indicating the presence of multiple conformational states (Figs. 4*B* and S9). Furthermore, 50 to 60% of the mutant G4 molecules exhibited frequent state transitions, correlating with their lower melting temperatures in Figure 3*D* (*Tm* = 55 °C, 52 °C, 50 °C, and 45 °C for 3T, 5A, 10C, and 16T, respectively). These findings suggest that SNVs in G-runs increase cMyc-G4 structural flexibility, destabilizing its native conformation and enhancing interconversion between distinct folding states. This compromised structural stability may have functional implications for G4-mediated gene regulation.

### SNVs in G-runs disrupt canonical G4 folding, leading to nonstandard architectures

**Figure 5:**
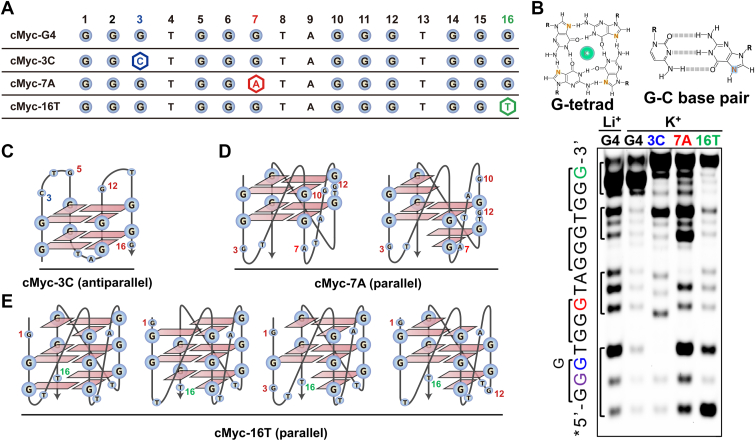
**The presumed structural organization form of selective G4 mutants detected by DMS-footprinting.***A*, mutation sites of selected G4 mutants. *B*, DMS-footprinting of 0.2 μM cMyc-G4 and three mutants in 100 mM KCl and/or 100 mM LiCl. Protected guanines indicate involvement in G-tetrad formation. *C–E*, proposed structural models for mutants 3C, 7A, and 16T, based on CD profiles and DMS-footprinting patterns. These models depict noncanonical features such as G-vacancies, bulges, and alternative loop configurations.

While the increased structural dynamics and state transitions induced by G-run SNVs reflect a destabilized G4 landscape, certain mutations may further disrupt the canonical G4 folding, leading to the emergence of nonstandard conformations. To investigate these noncanonical G4 configurations induced by SNVs, we first employed AlphaFold 3 for structural modeling. Due to the low prediction confidence of the nonstandard G4 sequences, we employed dimethyl sulfate (DMS) footprinting to experimentally identify the guanines involved in G-tetrad formation (Figs. 5, *A* and *B*, S10). DMS modification revealed that while the wild-type cMyc-G4 was fully protected from cleavage in 100 mM KCl, indicating a compact and well-folded structure, SNV-containing mutants displayed increased guanine accessibility, suggesting looser, less stable G4 folding.

Based on the footprinting patterns and CD profiles, we proposed possible structural models for three representative mutants (Fig. 5, *C*–*E*), which predominantly adopt nonstandard G4 conformations. These findings align with reports of partially folded G4 structures, such as G-triplexes, G-hairpins, and configurations featuring bulges, terminal G-triads, or 0-nt loops (3, 29, 30, 31).

First, the 3C: Forms an antiparallel conformation, with guanines at positions 5, 12, and 16 excluded from G-tetrad formation. The Gs involved in forming G-tetrads are likely underlined as: GGCTGGGTAGGGTGGG (Figs. 5*B*, S10). This configuration supports an antiparallel strand orientation, as illustrated in Figure 5*C*.

Second, the 7A: Adopts a parallel topology. Significant modifications occur at positions 3, 10, and 12, suggesting their involvement in loop regions GGGTGGATAGGGTGGG (Figs. 5*B*, S10). The sequence likely includes a 2-nt bulge between the 11th and 14th Gs (Fig. 5*D*, left panel). Alternatively, it could include G-vacancies (Fig. 5*D*, right panel).

Third, the 16T: the first guanine is not involved in Hoogsteen hydrogen bonding, while the third and twelfth guanines display progressively weaker cleavage bands (Figs. 5*B* and S10). It may adopt a parallel conformation featuring G-triads and G-hairpins (Fig. 5*E*). Panel 1 to 2: a G-vacancy forms with the first G excluded from a G-tetrad (GGGTGGGTAGGGTGGT). Panel 3: both the first and third Gs are excluded, leading to incomplete G-tetrads (GGGTGGGTAGGGTGGT). Panel 4: exclusion of the 1st and 12th Gs results in similar incomplete G4 structures (GGGTGGGTAGGGTGGT).

Together, these findings show that SNVs in G-runs do not merely remove a single G-tetrad but can generate diverse noncanonical G4 structures, such as G-vacancies, bulges, and extended loops.

### SNV-induced structural heterogeneity extends to other regulatory G4s

To investigate whether SNV-induced structural heterogeneity is a general feature of regulatory G4s beyond *cMyc*, we examined the insulin-linked polymorphic region G4 (ILPR-G4) (32). First, the representative loop mutant L3 with a T-to-G substitution was tested. CD spectra and gel-filtration (Fig. S11, *A* and *B*) showed that L3 maintains an oligomeric state comparable to the wild type but exhibits increased thermal stability. Moreover, its folding topology shifts from a hybrid form to predominantly parallel with a minor antiparallel fraction. DMS-footprinting (Fig. S11*C*) revealed that, in wild-type ILPR, guanines within the TGT loops are not engaged in Hoogsteen bonding. In contrast, in L3, the guanines in the GGT and TGT motifs, which are expected to reside in the loops, show markedly decreased reactivity. This suggests that, in certain conformations, nearly all guanines are engaged in Hoogsteen interactions, effectively shortening the loops and driving a conformational transition.

We next examined randomly selected G-run mutants ILPR-3C and ILPR-23A. CD spectra (Fig. S11*D*) indicated that both primarily adopt a parallel topology with a minor antiparallel fraction. For ILPR-3C, weak cleavage at guanines in the four G-runs suggests the contributions of 2, 3, 3, and 4 guanines to Hoogsteen bonding (Fig. S11*E*). This pattern implies that the guanine at position 25 (3′ end) may compensate for a vacancy in the first G-run, yielding a noncanonical three-layered G-quadruplex (Fig. S11*F*). For ILPR-23A, DMS-footprinting showed that G at position 8 remains unprotected, indicating its exclusion from Hoogsteen bonding, while the G in the last TGT loop exhibits reduced reactivity, suggesting partial involvement in G-tetrad formation. These observations support the presence of a top-layer G-triad with bulges.

Collectively, these results demonstrate that SNV-induced structural heterogeneity and loop mutation-mediated stabilization are not unique to the *cMyc* promoter but can also be reproduced in other regulatory G4s.

### Mechanistic consequences of SNVs on DNA replication

**Figure 6:**
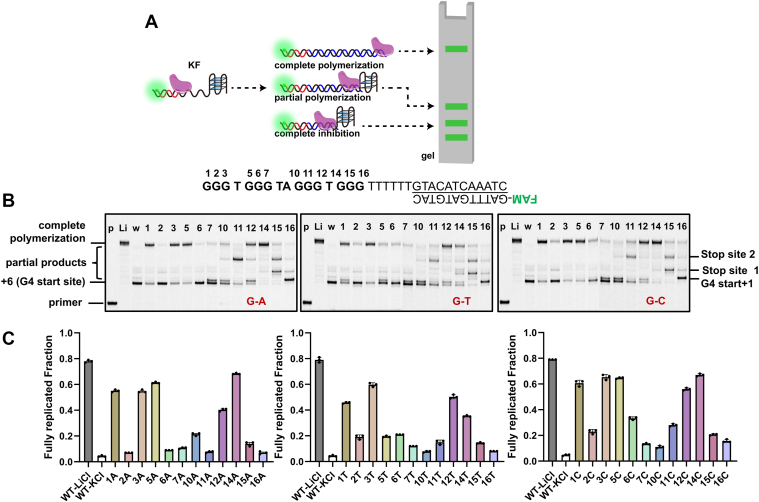
**Impact of G-run SNVs on DNA synthesis.***A*, schematic of the KF polymerase stop assay. Partial polymerization may result from misfolded G4 structures or other conformations. *B*, representative gel images of polymerase stop assays performed with 500 nM wild-type cMyc-G4 or its G-run mutants in 100 mM KCl. Lanes: p, 12-nt primer; Li, WT sequence in 100 mM LiCl (G4-destabilizing control); w, WT sequence in 100 mM KCl; other lanes are labeled with the mutated guanine positions in the cMyc-G4 sequence. Replication outcomes are categorized as: full-length product, complete stalling (G4 start site), or partial synthesis with discrete pause sites (G4 start+1, Stop 1 and Stop 2). *C*, quantification of replication efficiency. The bar graph shows the fraction of full-length product for cMyc-G4 and each mutant, quantified from gel images in (*B*) and Figure S12. Data are presented as mean ± SD (n = 3 independent experiments).

Having established that G-run SNVs induce diverse noncanonical G4 conformations in both cMyc and ILPR sequences, we next sought to investigate how such structural alterations influence DNA replication. Given the well-characterized regulatory role of cMyc-G4 and availability of robust functional assays, we focused our polymerase stop assays on the cMyc system as a representative model (Fig. 6*A*). A fluorescently labeled primer was annealed to a template containing the G4 sequence, positioned six nucleotides downstream of the duplex stem, enabling precise mapping of polymerase pausing within the G4 region.

Under our assay conditions, wild-type cMyc-G4 in 100 mM KCl induced almost complete polymerase stalling, confirming its strong inhibitory effect on DNA synthesis (Figs. 6*B*, S12). In contrast, the same sequence in 100 mM LiCl, which disfavors G4 formation, permitted efficient full-length synthesis, validating that the replication blockage was indeed G4-dependent. SNV-containing mutants displayed location-dependent effects on polymerase progression, which could be categorized into three distinct patterns: (i) complete polymerization, resembling the LiCl control; (ii) complete inhibition, similar to the wild-type G4; and (iii) partial synthesis, characterized by diffuse bands or discrete pause sites.

Among the partial products, two predominant stop sites were consistently observed, designated as stop site 1 and stop site 2, which likely correspond to the positions of the second and third G-runs relative to the G4 start site (Fig. 6*B*). This pattern suggests that even destabilized G4 mutants retain sufficient structural integrity to impede the polymerase at specific, structurally defined positions. Notably, mutations at the 16th guanine position, while retaining a parallel-stranded topology but exhibiting reduced thermal stability (*Tm* ∼40–50 °C; Fig. 3*D*), predominantly yielded single-base elongation products (Fig. 6*B*). This observation aligns with DMS-footprinting results (Fig. 5*B*), in which the first guanine remained unprotected, indicating disruption of a tetrad and partial destabilization of the G4 structure.

We further quantified the proportion of fully extended product for each mutant to assess replication efficiency (Fig. 6*C*). The efficiency correlated with both the mutation position and the substituting nucleotide, rather than thermostability alone.

These findings highlight that SNVs within G4 sequences can modulate polymerase progression by altering G4 conformation, stability, and structural dynamics. Given the role of G4s in genome regulation, our study underscores the potential impact of SNV-induced G4 variations on DNA metabolism, particularly in replication-associated mutagenesis and the maintenance of genome integrity.

### SNVs modulate the transcriptional regulation of *cMyc* promoter G4 in a diverse manner

**Figure 7:**
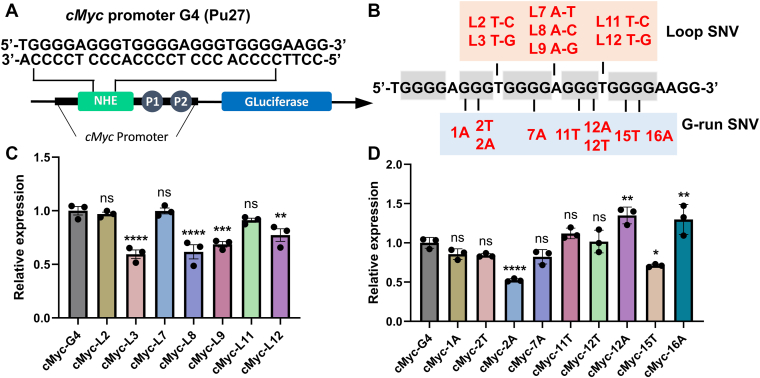
**SNVs differentially modulate the transcriptional regulation of the *cMyc* promoter G4.***A*, schematic of the *Gaussia* luciferase (GLuc) reporter assay. The *cMyc* promoter sequence containing wild-type (WT) or mutant Pu27 were cloned upstream of the luciferase gene in the pGLuc-Basic vector. *B*, sequences of the 17 SNV mutants analyzed, including 7 loop mutants (L2, L3, L7, L8, L9, L11, L12) and 9 G-run mutants (1A, 2T, 2A, 7A, 11T, 12T, 12A, 15T, 16A). *C*, relative GLuc activity of loop mutants. Data were normalized to the WT level (set to 1). Stabilizing loop SNVs (L3, L8, L9, L12) significantly suppress gene expression, while others (L2, L7, L11) show minimal changes. *D*, relative GLuc activity of G-run mutants. Destabilizing G-run SNVs produce heterogeneous transcriptional effects: some increase expression (12A, 16A), others decrease it (2A, 15T), and several show little change. Data in (*C*) and (*D*) represent mean ± SD from at least three independent biological replicates. Statistical significance was determined by one-way ANOVA with Dunnett’s *post hoc* test *versus* WT (∗*p* < 0.05, ∗∗*p* < 0.01, ∗∗∗*p* < 0.001, ∗∗∗∗*p* < 0.0001).

Given the critical role of G4 structures in transcriptional control, we next examined whether SNVs within the *cMyc* promoter G4 sequence affect gene expression. To evaluate their functional impact, we employed a *Gaussia* luciferase (GLuc) reporter assay in which the wild-type (WT) or mutant Pu27 sequences were cloned upstream of the luciferase gene (Fig. 7*A*). HeLa cells were transfected with these constructs, and GLuc activity was subsequently quantified 48 h later and normalized. Based on the SNVs identified in the dbSNP database (Fig. 1) and our biophysical studies, we designed and tested 16 mutants, encompassing 7 loop and 9 G-run variants (Fig. 7*B*).

For the 7 loop mutants, GLuc expression was predominantly reduced (Fig. 7*C*). In particular, L3, L8, L9, and L12 showed a 1/3–1/2 decrease in activity, which correlated with their increased *Tm* values (Fig. S2, *A* and *B*). By contrast, L2, L7, and L11 exhibited minimal changes in GLuc expression, consistent with their modest *Tm* alterations. Since loop SNVs retain the same overall structure as the WT (Figs. 2*E*, S5), significant changes in *Tm* directly lead to marked variations in GLuc expression. Notably, L3 (rs1402286402 T-G), L9 (rs1296869403 A-G), and L12 (rs13250910 T-G), the most frequent SNVs identified with MAFs of 20.6%, 21.2%, and 19.931%, significantly suppressed downstream gene expression. These results align with the long-standing view that the *cMyc* promoter G4 functions as a transcriptional repressor.

In contrast, the effects of G-run SNVs were more heterogeneous (Fig. 7*D*). Although all G-run mutants displayed decreased *Tm* values (Fig. 3*D*), their transcriptional impact varied: some increased GLuc expression, others decreased it, and several showed little change. For example, mutants 12A and 16A elevated expression by ∼one-third, whereas 2A and 15T reduced expression by half and one-third, respectively. No straightforward correlation emerged between *Tm*, conformational changes, and expression outcomes. We speculate that G-run SNVs generate non-canonical G4 architectures (Fig. 5) with high structural dynamics (Fig. 4), and that their regulatory effects depend on specific structural features influencing interactions with transcription factors, polymerases, or other proteins.

In summary, SNVs within the *cMyc* promoter G4 can significantly alter transcriptional output. The underlying mechanisms are multifaceted: stabilizing the G4 without changing its topology generally suppresses expression, whereas destabilization does not consistently relieve repression and may, in some cases, enhance it.

## Discussion

In this study, we systematically investigated the impact of SNVs on the structure and function of the *cMyc* promoter G4 using an extensive set of mutants. Our bioinformatic analyses of the dbSNP database revealed that the Pu27 G4 region is a mutational hotspot, with SNVs distributed across both loop regions and G-runs. Loop bases display higher minor allele frequencies, often with substitutions to guanine, whereas G-runs are relatively conserved, reflecting the purifying selection. Building on these observations, our biophysical analyses demonstrate that *cMyc* promoter G4 is remarkably tolerant to variations in both loop regions and G-runs, contrary to the prevailing belief that SNVs inherently destabilize G4 structures (33, 34, 35). This observation reveals a more complex interplay between SNVs and G4 stability than previously appreciated.

### SNVs in G-runs drive structural polymorphism across diverse G4s

Rather than simply eliminating a G-tetrad, SNVs in G-runs can give rise to a spectrum of noncanonical G4 structures, including G-triads, G-G base pairs, G-vacancies, bulges, and even 0-nucleotide loops (3, 29, 30, 31). These structural variations often coexist dynamically, as demonstrated by our DMS-footprinting (Fig. 5) and single-molecule FRET assays (Fig. 4), which reveal state transitions among multiple conformers. Importantly, this structural heterogeneity is not unique to the *cMyc* promoter but also occurs in other regulatory G4s such as ILPR (Fig. S11, *D* and *F*), indicating a widespread genomic phenomenon. Based on our DMS-footprinting and CD analyses, we proposed preliminary structural models for these mutant G4s, identifying which guanines participate in G-tetrads and assessing their overall topology. In the future, it will be important to determine the three-dimensional structures of key mutant G4s using high-resolution experimental approaches. Together, our findings suggest that SNV-induced structural heterogeneity is a widespread phenomenon that extends beyond cMyc-G4, potentially affecting various genomic G4s. This structural polymorphism induced by SNVs may provide a mechanism for fine-tuning G4-mediated regulatory functions, potentially influencing transcription, replication, and genome stability in a context-dependent manner.

### Loop SNVs stabilize G4s by enhancing Hoogsteen bonding

While most studies have focused on SNVs within G-runs, our findings reveal that variations in loop regions, where substitution frequencies are often higher, can also significantly alter G4 stability. Surprisingly, certain loop SNVs enhance G4 thermostability, likely through additional hydrogen bonds and π–π stacking interactions that reinforce the overall structure (Fig. 3*E*). This stabilizing effect is further exemplified by the ILPR G4 loop mutant L3 (Fig. S11, A–C), in which nearly all guanines engage in Hoogsteen bonding, effectively shortening the loops and triggering a conformational transition. These observations highlight the nuanced and context-dependent roles of loop SNVs in shaping G4 structural landscapes. Together, our findings broaden the understanding of SNV impacts on G4 structure-function relationships across the genome and underscore the importance of considering both disruptive and stabilizing SNVs in genomic studies.

### Functional and biological implications of SNV-induced G4 variants

The structural polymorphism induced by SNVs extends beyond simple conformational shifts and has functional consequences. Our study provides a multilevel mechanistic basis for the associations observed in genome-wide analyses, which have linked G4-SNVs to changes in gene expression and transcriptional regulation (6, 10, 11, 12, 13, 14, 15, 16, 36). At the DNA level, our polymerase-stop assays (Fig. 6) demonstrate that different G4 variants impede polymerase progression to varying degrees, producing DNA fragments of distinct lengths. Such variations in replication efficiency can influence both DNA metabolism and, indirectly, transcription. Our reporter assays (Fig. 7) directly connect these structural and replicative perturbations to transcriptional outcomes. We found that stabilizing loop SNVs, which maintain the overall G4 topology, consistently suppress promoter-driven gene expression. This supports the canonical view of the *cMyc* promoter G4 as a transcriptional repressor, a role established in synthetic reporter systems and through G4-stabilizing ligands.

However, the regulatory role of G4s is context-dependent and more complex than a simple repressor. A recent CRISPR study disrupting G4 folding at the endogenous *cMyc* locus showed that the G4 structure can act as a positive regulator, facilitating recruitment of transcription factors and chromatin modifiers to establish an active chromatin state (21). Our findings on destabilizing G-run SNVs, which produce heterogeneous transcriptional effects, sometimes relieving repression, in other contexts enhancing it, align with this paradigm. The adoption of non-canonical architectures and increased structural dynamics (Figs. 4 and 5) likely remodels the repertoire of interacting regulatory proteins, thereby shifting transcriptional output from repression toward activation. Collectively, our findings demonstrate that even subtle SNV-induced changes in G4 topology can have significant and diverse regulatory consequences, impacting gene expression through integrated effects that are contingent upon the resulting structure and its specific molecular interactions.

In conclusion, our study expands the understanding of G4 structural diversity and highlights the functional implications of G4-SNV interactions. These variations in nucleic acid secondary structures may exert a greater impact on biological processes than positional variants elsewhere in the genome. The ability of G4 structures to adapt to SNVs reflects their functional and pathological versatility, offering new perspectives for genetic variation research. Furthermore, these findings have potential applications in health risk assessment, disease diagnosis, and the development of targeted therapies.

## Experimental procedures

### Oligonucleotide and DNA preparation

All oligonucleotides were purchased from Sangon Biotech, with their sequences and labeling details listed in Table S1. Loop and G-run mutations were designated as Ln and nN, respectively (n: position, N: A, C, or T). DNA samples were annealed by heating at 95 °C for 5 min, followed by slow cooling to room temperature (∼7 h) in a uniform buffer to ensure proper G4 folding.

### Circular dichroism (CD) spectroscopy

CD spectra and melting curves were recorded on a Chirascan V100 spectrometer (Applied Photophysics, UK) using a 1-mm path length quartz cuvette. Annealed 4 μM oligonucleotides were dissolved in 25 mM Tris-HCl (pH 7.5) with 100 mM KCl, unless otherwise specified. Spectra were collected over a wavelength range of 220 to 320 nm with a 1.0 nm step size at 25 °C. The melting experiments were performed by heating the annealed samples from 25 °C to 95 °C with 2 °C step (1.0 °C/min), and the temperature was maintained stable for 1 min during recording. *Tm* was determined from the change of ellipticity at 263 nm or 293 nm.

### Native polyacrylamide gel electrophoresis (PAGE)

Native PAGE was performed to assess G4 assembly. FAM-labeled G4 DNA (4 μM) was annealed in 25 mM Tris-HCl (pH 7.5) with 100 mM KCl. Samples (100 nM DNA) were loaded onto 15% PAGE in 1×TAE buffer and electrophoresed at 120 V for 1 h. Bands were visualized using a ChemiDocMP imaging system (Bio-Rad).

### Gel filtration chromatography

Gel filtration was conducted on an AKTA purifier (GE Healthcare) with a Superdex 200 Increase 10/300 Gl column (Cytiva). The column was equilibrated with 25 mM Tris-HCl (pH 7.5) and 100 mM KCl at 0.2 ml/min. Annealed DNA (4 μM, 100 μl) was loaded and eluted at the same flow rate while monitoring absorbance at 260 nm.

### Nuclear magnetic resonance (NMR) spectroscopy

NMR experiments were performed using a Bruker Avance III 500 MHz spectrometer (Bruker). DNA (300 μM) was dissolved in 25 mM Tris-HCl (pH 7.5) buffer (D_2_O/H_2_O, 1:9) with 100 mM KCl and annealed. 1D ^1^H NMR spectra were recorded at 25 °C with 3600 scans.

### Fluorescent probe assay

ISCH-1, a dual-colorimetric and fluorescent probe, was used to detect G4 structures (37). DNA (1 μM) was annealed in 25 mM Tris-HCl (pH 7.5) with 100 mM KCl. Emission spectra (600–800 nm, λ_ex_ = 560 nm) were recorded at 25 °C on a SpectraMax iD5 microplate reader (Molecular Devices). ISCH-1 was kindly provided by Prof. Jia-Heng Tan (Sun Yat-sen University).

### Single-molecule FRET (smFRET) and data analysis

The smFRET experiments followed a previously described protocol (38). Annealed DNA (50 pM) in 25 mM Tris-HCl (pH 7.5) with varying KCl concentrations (20–100 mM) was immobilized in chambers pre-coated with streptavidin (10 μg/ml). An oxygen scavenging system was used to minimize free DNA and ensure stable conditions. Data were collected at 22 °C with a 100 ms exposure time. FRET efficiency was calculated as E = I_A_/(I_D_ + I_A_), where I_D_ and I_A_ are donor and acceptor intensities, respectively. Analysis included at least 300 traces using MATLAB scripts, with fitting in Origin 8.0.

### Dimethyl sulfate (DMS) footprinting

The DMS-footprinting assays followed a previous protocol (29). Oligonucleotides (0.2 μM), labeled at the 5′-end with a FAM fluorophore (Table S1), were annealed in 25 mM Tris-HCl (pH 7.5) buffer with 100 mM LiCl or KCl and 0.5 μM EDTA. Products were treated with 5% DMS for 4 min at room temperature, and the reaction was stopped with an equal volume of stop buffer (3 M sodium acetate, 0.1 M β-mercaptoethanol, and 1 mg/ml spermidine DNA). After chloroform extraction and ethanol precipitation, substrates were cleaved with 10% piperidine for 30 min at 90 °C. Following another round of purification, samples were dissolved in 8 M urea, heated at 95 °C for 5 min, cooled on ice for 10 min, and loaded onto 20% PAGE with 7 M urea. The gel was imaged using the ChemiDocMP system. All experiments were repeated independently at least three times.

### Polymerase stop assay

The polymerase stop assay was performed as described (39). A 1:1 mixture of a FAM-labeled 12-nt primer and a G4-forming DNA template was annealed in 25 mM Tris-HCl (pH 7.5) with 100 mM KCl. The reaction included 500 nM annealed substrates, 100 nM Klenow Fragment (KF), 100 μM dNTPs, and 6 mM MgCl_2_, incubated at 22 °C for 10 min. The reaction was stopped with 8 M urea, heated at 95 °C for 10 min, and analyzed on 20% PAGE with 8 M urea. Gels were imaged using a ChemiDocMP system. Band intensities were quantified using ImageJ. The integrated density (IntDen) was measured by drawing ROIs that covered either the entire lane or fully extended product bands, respectively. The fraction of a fully extended band was calculated as its IntDen divided by the total IntDen of the corresponding lane. All experiments were performed in at least three independent replicates.

### *Gaussia* luciferase reporter assay

The reporter plasmid pGLuc-Basic, containing the *cMyc* promoter, was a generous gift from Dr Wei-Wei Huang (Northwest A&F University, China). Site-directed mutagenesis of the G4-forming Pu27 region was performed using homologous recombination to generate all SNV mutants. HeLa cells, confirmed to be free of *mycoplasma* contamination, were transfected with either wild-type or mutant constructs, and *Gaussia* luciferase (GLuc) activity in the culture medium was measured 48 h post-transfection using the Secrete-Pair *Gaussia* Luciferase Assay Kit (GeneCopoeia).

To minimize potential variability, all plasmids were identical in vector backbone, reporter cassette, and selection marker, differing only by a single nucleotide within the Pu27 sequence. All constructs were prepared in the same batch, purified and quantified by the same method, and transfected at equal DNA concentrations under identical conditions (cell density, reagent, and ratio) by the same operator. Each construct was tested in at least three biological replicates (*i.e.*, three separately seeded, transfected, and measured cell cultures; individual data points shown), and the experiments were independently repeated multiple times to confirm reproducibility. Raw luminescence values were normalized to the wild-type control, which was set to 1. Statistical significance was evaluated by one-way ANOVA followed by Dunnett’s multiple comparisons test, with all mutants compared to WT. Data are presented as mean ± SD

### Alphafold3 structural prediction

The sequences corresponding to wild-type and mutant cMyc-G4 constructs were input into AlphaFold3 together with two K^+^, and predictions were performed using the default nucleotide modeling parameters. Structural confidence was assessed using the predicted Local Distance Difference Test (pLDDT) score, with values above 90 considered highly reliable.

## Data availability

All data presented in this study are available upon request.

## Supporting information

This article contains supporting information.

## Conflict of Interest

The authors declare that they do not have any conflicts of interest with the content of this article.
