# Supplementary material for: Mutational landscape of the G-quadruplex DNA in cMyc proto-oncogene promoter: Insights into the structural polymorphism and transcriptional regulation
Source: J Biol Chem. 2025 Nov 4;301(12):110897. doi: 10.1016/j.jbc.2025.110897 (PMC12704285; doi:10.1016/j.jbc.2025.110897)
Supplement: Supporting information [file mmc1.pdf]

Table S1. DNA sequences used in this study.

Name	Sequence (5' to 3')	Description
	**sequences of DNA for CD, CD-melting, Gel filtration, NMR, fluorescent probe** [a]	
cMyc-G4	GGG T GGG TA GGG T GGG	**parallel**
cMyc-L1	GGG A GGG TA GGG T GGG	parallel
cMyc-L2	GGG C GGG TA GGG T GGG	parallel
cMyc-L3	GGG G GGG TA GGG T GGG	parallel
cMyc-L4	GGG T GGG AA GGG T GGG	parallel
cMyc-L5	GGG T GGG CA GGG T GGG	parallel
cMyc-L6	GGG T GGG GA GGG T GGG	parallel
cMyc-L7	GGG T GGG TT GGG T GGG	Parallel
cMyc-L8	GGG T GGG TC GGG T GGG	parallel
cMyc-L9	GGG T GGG TG GGG T GGG	parallel
cMyc-L10	GGG T GGG TA GGG A GGG	parallel
cMyc-L11	GGG T GGG TA GGG C GGG	parallel
cMyc-L12	GGG T GGG TA GGG G GGG	parallel
cMyc-1A	AGG T GGG TA GGG T GGG	parallel
cMyc-2A	GAG T GGG TA GGG T GGG	parallel
cMyc-3A	GGA T GGG TA GGG T GGG	parallel + antiparallel
cMyc-5A	GGG T AGG TA GGG T GGG	parallel
cMyc-6A	GGG T GAG TA GGG T GGG	parallel
cMyc-7A	GGG T GGA TA GGG T GGG	parallel
cMyc-10A	GGG T GGG TA AGG T GGG	parallel
cMyc-11A	GGG T GGG TA GAG T GGG	parallel
cMyc-12A	GGG T GGG TA GGA T GGG	parallel
cMyc-14A	GGG T GGG TA GGG T AGG	antiparallel
cMyc-15A	GGG T GGG TA GGG T GAG	parallel
cMyc-16A	GGG T GGG TA GGG T GGA	parallel
cMyc-1C	CGG T GGG TA GGG T GGG	parallel
cMyc-2C	GCG T GGG TA GGG T GGG	parallel
cMyc-3C	GGC T GGG TA GGG T GGG	antiparallel
cMyc-5C	GGG T CGG TA GGG T GGG	parallel
cMyc-6C	GGG T GCG TA GGG T GGG	parallel
cMyc-7C	GGG T GGC TA GGG T GGG	parallel
cMyc-10C	GGG T GGG TA CGG T GGG	parallel
cMyc-11C	GGG T GGG TA GCG T GGG	parallel
cMyc-12C	GGG T GGG TA GGC T GGG	parallel
cMyc-14C	GGG T GGG TA GGG T CGG	antiparallel
cMyc-15C	GGG T GGG TA GGG T GCG	parallel
cMyc-16C	GGG T GGG TA GGG T GGC	parallel
cMyc-1T	TGG T GGG TA GGG T GGG	parallel

cMyc-2T	GTG T GGG TA GGG T GGG	parallel
cMyc-3T	GGT T GGG TA GGG T GGG	parallel + antiparallel
cMyc-5T	GGG T TGG TA GGG T GGG	parallel
cMyc-6T	GGG T GTG TA GGG T GGG	parallel
cMyc-7T	GGG T GGT TA GGG T GGG	parallel
cMyc-10T	GGG T GGG TA TGG T GGG	parallel
cMyc-11T	GGG T GGG TA GTG T GGG	parallel
cMyc-12T	GGG T GGG TA GGT T GGG	parallel
cMyc-14T	GGG T GGG TA GGG T TGG	parallel + antiparallel
cMyc-15T	GGG T GGG TA GGG T GTG	parallel
cMyc-16T	GGG T GGG TA GGG T GGT	parallel

sequences of DNA for smFRET [a, b]

Stem	TCTCC**T(iCy5)**ATTTCCTATTACCGGTGCCACGC-**Biotin**
d_{29}cMyc-G4	GCGTGGCACCGGTAATAGGAAATAGGAGATT**GGGTGGGTAGGGTGGGTT(iCy3)**
d_{29}cMyc-3T	GCGTGGCACCGGTAATAGGAAATAGGAGATT**GGTTGGGTAGGGTGGGTT(iCy3)**
d_{29}cMyc-5A	GCGTGGCACCGGTAATAGGAAATAGGAGATT**GGGTAGGTAGGGTGGGTT(iCy3)**
d_{29}cMyc-10C	GCGTGGCACCGGTAATAGGAAATAGGAGATT**GGGTGGGTACGGTGGGTT(iCy3)**
d_{29}cMyc-16T	GCGTGGCACCGGTAATAGGAAATAGGAGATT**GGGTGGGTAGGGTGGTTT(iCy3)**

sequences of DNA for DMS-footprinting and Native PAGE [a]

FAM-cMyc-G4	**FAM**-GGGTGGGTAGGGTGGG
FAM-cMyc-1T	**FAM**-TGGTGGGTAGGGTGGG
FAM-cMyc-2T	**FAM**-GTGTGGGTAGGGTGGG
FAM-cMyc-3T	**FAM**-GGTTGGGTAGGGTGGG
FAM-cMyc-3C	**FAM**-GGCTGGGTAGGGTGGG
FAM-cMyc-7A	**FAM**-GGGTGGATAGGGTGGG
FAM-cMyc-16T	**FAM**-GGGTGGGTAGGGTGGT
FAM-G4TG4	**FAM**-GGGTGGGTAGGGTGGG**T**GGGTGGGTAGGGTGGG
FAM-G4T3G4	**FAM**-GGGTGGGTAGGGTGGG**TTT**GGGTGGGTAGGGTGGG
FAM-ILPR	**FAM**-GGGGTGTGGGGACAGGGGTGTGGGG
FAM-ILPR-3C	**FAM**-GGCGTGTGGGGACAGGGGTGTGGGG
FAM-ILPR-23A	**FAM**-GGGGTGTGGGGACAGGGGTGTGGGG
FAM-ILPR-L3	**FAM**-GGGGGGTGGGGACAGGGGTGTGGGG

sequences of DNA for DNA polymerase stop assay [a, b]

FAM-12nt	**FAM**-GATTTGATGTAC
cMyc-G4-s6d12	**GGGTGGGTAGGGTGGG**TTTTTTGTACATCAAATC
cMyc-1A-s6d12	**AGGTGGGTAGGGTGGG**TTTTTTGTACATCAAATC
cMyc-2A-s6d12	**GAGTGGGTAGGGTGGG**TTTTTTGTACATCAAATC
cMyc-3A-s6d12	**GGATGGGTAGGGTGGG**TTTTTTGTACATCAAATC
cMyc-5A-s6d12	**GGGTAGGTAGGGTGGG**TTTTTTGTACATCAAATC
cMyc-6A-s6d12	**GGGTGAGTAGGGTGGG**TTTTTTGTACATCAAATC

cMyc-7A-s6d12	**GGGTGGATAGGGTGGG**TTTTTTGTACATCAAATC
cMyc-10A-s6d12	**GGGTGGGTAAGGTGGG**TTTTTTGTACATCAAATC
cMyc-11A-s6d12	**GGGTGGGTAGAGTGGG**TTTTTTGTACATCAAATC
cMyc-12A-s6d12	**GGGTGGGTAGGATGGG**TTTTTTGTACATCAAATC
cMyc-14A-s6d12	**GGGTGGGTAGGGTAGG**TTTTTTGTACATCAAATC
cMyc-15A-s6d12	**GGGTGGGTAGGGTGAG**TTTTTTGTACATCAAATC
cMyc-16A-s6d12	**GGGTGGGTAGGGTGGA**TTTTTTGTACATCAAATC
cMyc-1C-s6d12	**CGGTGGGTAGGGTGGG**TTTTTTGTACATCAAATC
cMyc-2C-s6d12	**GCGTGGGTAGGGTGGG**TTTTTTGTACATCAAATC
cMyc-3C-s6d12	**GGCTGGGTAGGGTGGG**TTTTTTGTACATCAAATC
cMyc-5C-s6d12	**GGGTCGGTAGGGTGGG**TTTTTTGTACATCAAATC
cMyc-6C-s6d12	**GGGTGCGTAGGGTGGG**TTTTTTGTACATCAAATC
cMyc-7C-s6d12	**GGGTGGCTAGGGTGGG**TTTTTTGTACATCAAATC
cMyc-10C-s6d12	**GGGTGGGTACGGTGGG**TTTTTTGTACATCAAATC
cMyc-11C-s6d12	**GGGTGGGTAGCGTGGG**TTTTTTGTACATCAAATC
cMyc-12C-s6d12	**GGGTGGGTAGGCTGGG**TTTTTTGTACATCAAATC
cMyc-14C-s6d12	**GGGTGGGTAGGGTCGG**TTTTTTGTACATCAAATC
cMyc-15C-s6d12	**GGGTGGGTAGGGTGCG**TTTTTTGTACATCAAATC
cMyc-16C-s6d12	**GGGTGGGTAGGGTGGC**TTTTTTGTACATCAAATC
cMyc-1T-s6d12	**TGGTGGGTAGGGTGGG**TTTTTTGTACATCAAATC
cMyc-2T-s6d12	**GTGTGGGTAGGGTGGG**TTTTTTGTACATCAAATC
cMyc-3T-s6d12	**GGTTGGGTAGGGTGGG**TTTTTTGTACATCAAATC
cMyc-5T-s6d12	**GGGTTGGTAGGGTGGG**TTTTTTGTACATCAAATC
cMyc-6T-s6d12	**GGGTGTGTAGGGTGGG**TTTTTTGTACATCAAATC
cMyc-7T-s6d12	**GGGTGGTTAGGGTGGG**TTTTTTGTACATCAAATC
cMyc-10T-s6d12	**GGGTGGGTATGGTGGG**TTTTTTGTACATCAAATC
cMyc-11T-s6d12	**GGGTGGGTAGTGTGGG**TTTTTTGTACATCAAATC
cMyc-12T-s6d12	**GGGTGGGTAGGTTGGG**TTTTTTGTACATCAAATC
cMyc-14T-s6d12	**GGGTGGGTAGGGTTGG**TTTTTTGTACATCAAATC
cMyc-15T-s6d12	**GGGTGGGTAGGGTGTG**TTTTTTGTACATCAAATC
cMyc-16T-s6d12	**GGGTGGGTAGGGTGGT**TTTTTTGTACATCAAATC

[a] Mutated nucleotides are marked with red color.

[b] The underlined sequence is the complementary region.

Table S2. The SNV information of Pu27 in *cMyc* proto-oncogene promoter.

Variation ID	Variation type	Alleles	Minor allele frequency	Genome location	
rs1409012960	SNV, length 1	T/G/C	G: 6.727%(ALFA)；<br>G:2.466%(Korea1K)；<br>G:9.78%(KOREAN);<br>C:0.013% (GnomAD_exomes)	NC_00008.11@127735954	NC_00008.10@128748200
rs1813577733	SNV, length 1	G/A	A: 0.0004%（TOPMed）	NC_00008.11@127735952	NC_00008.10@128748198
rs1308963210	SNV, length 1	G/T/A	T:0.0026%(TOPMed)<br>A:0.0013%(gnomAD Genomes)	NC_00008.11@127735951	NC_00008.10@128748297
rs1222390996	SNV, length 1	A/G/C	G:15.955%(KOREAN)<br>C: not available	NC_00008.11@127735949	NC_00008.10@128748295
rs1813577483	SNV, length 1	G/A	A:0.0004%（TOPMed）	NC_00008.11@127735948	NC_00008.10@128748294
rs1285789760	SNV, length 1	G/T/A	A: 0.0015%（TOPMed）<br>T: not available	NC_00008.11@127735947	NC_00008.10@128748193
rs1402286402	SNV, length 1	T/G/C	G:20.6%(KOREAN)<br>G:13.43%(ALFA)<br>G:3.66%(TOMMO)<br>C: not available	NC_00008.11@127735945	NC_00008.10@128748291
rs928467627	SNV, length 1	G/A	A: 0.0004%（TOPMed）	NC_00008.11@127735942	NC_00008.10@128748288
rs1296869403	SNV, length 1	A/T/G/C	T: not available<br>G:21.2%(KOREAN)<br>G:8.9%（Korea4K）<br>C:0.002%(gnomAD_exomes)	NC_00008.11@127735940	NC_00008.10@128748186
rs1044244227	SNV, length 1	G/T/A	T:0.0007%(gnomAD_exomes)<br>A:0.0066%(gnomAD_exomes)	NC_00008.11@127735937	NC_00008.10@128748283
rs13250910	SNV, length 1	T/G/C/A	G:6.395%(TOMMO)<br>G:19.931%(KOREAN)<br>A:0.002%(GnomAD_genomes)<br>C:not available	NC_00008.11@127735936	NC_00008.10@128748282
rs135539287	SNV, length 1	G/T	T: not available	NC_00008.11@127735934	NC_00008.10@128748280
rs1813576856	SNV, length 1	G/A	A:0.002%(GnomAD_genomes)	NC_00008.11@127735933	NC_00008.10@128748279
rs538014647	SNV, length 1	G/T/C/A	A: 0.02%(1000Genomes)<br>T,C: not available	NC_00008.11@127735932	NC_00008.10@128748278
rs1813576582	SNV, length 1	A/G/C	G:0.0021% (GnomAD_genomes)<br>C:0.0027% (GnomAD_exomes)	NC_00008.11@127735931	NC_00008.10@128748277
rs2130081304	SNV, length 1	A/T/C	T:0.1093%(1000Genomes_30X)<br>C:0.0517%(TOMMO)	NC_00008.11@127735930	NC_00008.10@128748276
rs948569170	SNV, length 1	G/A/C	A:0.0142%(ALFA)<br>A:0.0102%(TOPMED)<br>A:0.0013%(GnomAD_exomes)<br>C:0.0013%(TOMMO)	NC_00008.11@127735929	NC_00008.10@128748275

Allele frequency data sources:

ALFA – Allele Frequency Aggregator (NCBI);

Korea1K – Korean Reference Genome Project (1,000 genomes);

Korea4K – Korean Reference Genome Project (4,000 genomes);

KOREAN – aggregated Korean population studies;

TOMMO – Tohoku Medical Megabank Project (Japan);

gnomAD_exomes – Genome Aggregation Database (exome sequencing data);

gnomAD_genomes – Genome Aggregation Database (whole genome sequencing data);

TOPMed – Trans-Omics for Precision Medicine program;

1000Genomes – 1000 Genomes Project (global population reference);

1000Genomes_30X – High-coverage (30×) whole-genome sequencing dataset from the 1000 Genomes Project.

Table S3. The deletion and insertion mutation information of Pu27 in *cMyc* proto-oncogene promoter.

Variation ID	Variation type	Alleles	Minor allele frequency	Genome location	
rs2130081461	Insertion, length 0	/TG	Not available	NC_00008.11@127735940	NC_00008.10@128748186
rs1813576676	Insertion, length 0	/T/GT/C/GC/GGC/GA/GGA	Not available	NC_00008.11@127735932	NC_00008.10@128748178
rs1343043609	Deletion, length 1	A/	0.548%(Korea1K); 0.047% (1000Genomes_30X)	NC_00008.11@127735949	NC_00008.10@128748295
rs1373221626	Deletion, length 1	T/	0.548%(Korea1K); 0.062%(1000Genomes_30X)	NC_00008.11@127735945	NC_00008.10@128748291
rs1463847192	Deletion, length 1	A/	0.658%(Korea1K); 0.066%(TOMMO)	NC_00008.11@127735940	NC_00008.10@128748286
rs1354481566	Deletion, length 1	T/	0.659%(Korea1K); 0.234%(TOMMO)	NC_00008.11@127735936	NC_00008.10@128748182
rs2130081409	Deletion, length 5	AGGGT/	0.096%(TOMMO)	NC_00008.11@127735936	NC_00008.10@128748282
rs2130081357	Deletion, length 4	GGGG/	0.225%(TOMMO)	NC_00008.11@127735932	NC_00008.10@128748278
rs2130081450	Deletion-Insertion, length 3	GAG/G	0.043%(TOMMO)	NC_00008.11@127735939	NC_00008.10@128748185
rs2130081440	Deletion-Insertion, length 5	GGAGG/GG	0.107%(TOMMO)	NC_00008.11@127735938	NC_00008.10@128748284
rs1327195180	Deletion-Insertion, length 4	GGGG/GGG/GGGGG/GGGGGG/GGGGGGG	GGGGGGG 0.067%(ALFA)	NC_00008.11@127735932	NC_00008.10@128748278
rs1175897709	Deletion-Insertion, length 24	TGGGGAGGGTGGGGAGGGTGGGGA/ TGGGGAGGGTGGGGA/ TGGGGAGGGTGGGGAGGGTGGGGAGGGTGGGGA	Not available	NC_00008.11@127735931	NC_00008.10@128748277
rs1813576526	Deletion-Insertion, length 2	AA/AAA	0.129%(TOMMO)	NC_00008.11@127735930	NC_00008.10@128748276

Table S4. The additional Hoogsteen H-bonds and Pi-Pi stacking in cMyc-G4 and the loop mutants predicted by Alphafold3.

sequence	Name	Hoogsteen H-bond	Additional H-bond	Pi-Pi stacking
GGGTGGGTAGGGTGGG	Wild type	8×3		16
GGGAGGGTAGGGTGGG	L1	8×3		16+2
GGGCGGGTAGGGTGGG	L2	8×3		16+1
GGGGGGGTAGGGTGGG	L3	8×3		16+3
GGGTGGGAAGGGTGGG	L4	8×3		16+1
GGGTGGGCAGGGTGGG	L5	8×3		16
GGGTGGGGAGGGTGGG	L6	8×3	1	16
GGGTGGGTTGGGTGGG	L7	8×3		16
GGGTGGGTCGGGTGGG	L8	8×3		16+5
GGGTGGGTGGGGTGGG	L9	8×3	3	16+6
GGGTGGGTAGGGAGGG	L10	8×3		16
GGGTGGGTAGGGCGGG	L11	8×3		16+1
GGGTGGGTAGGGGGGG	L12	8×3		16

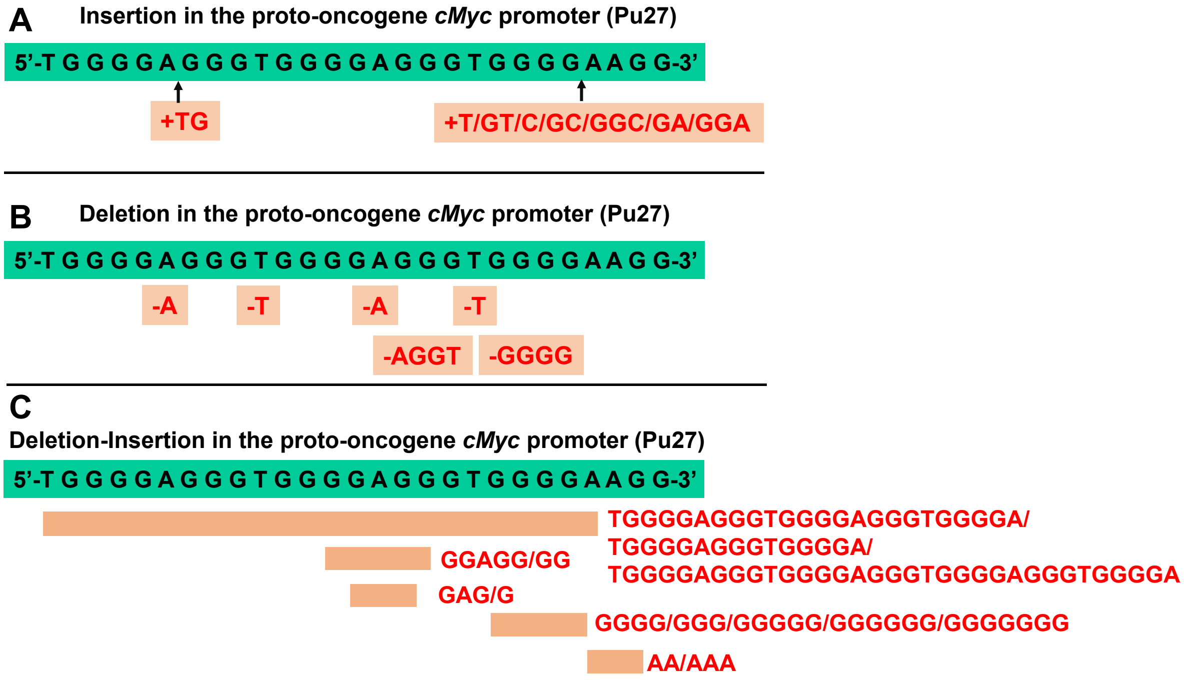

Figure S1. Indels and complex variants in the Pu27 element from dbSNP. (A) Insertions. **(B)** Deletions. **(C)** Deletion-insertion variants. All sequence alterations are mapped onto the reference Pu27 sequence, with variant alleles highlighted in red.

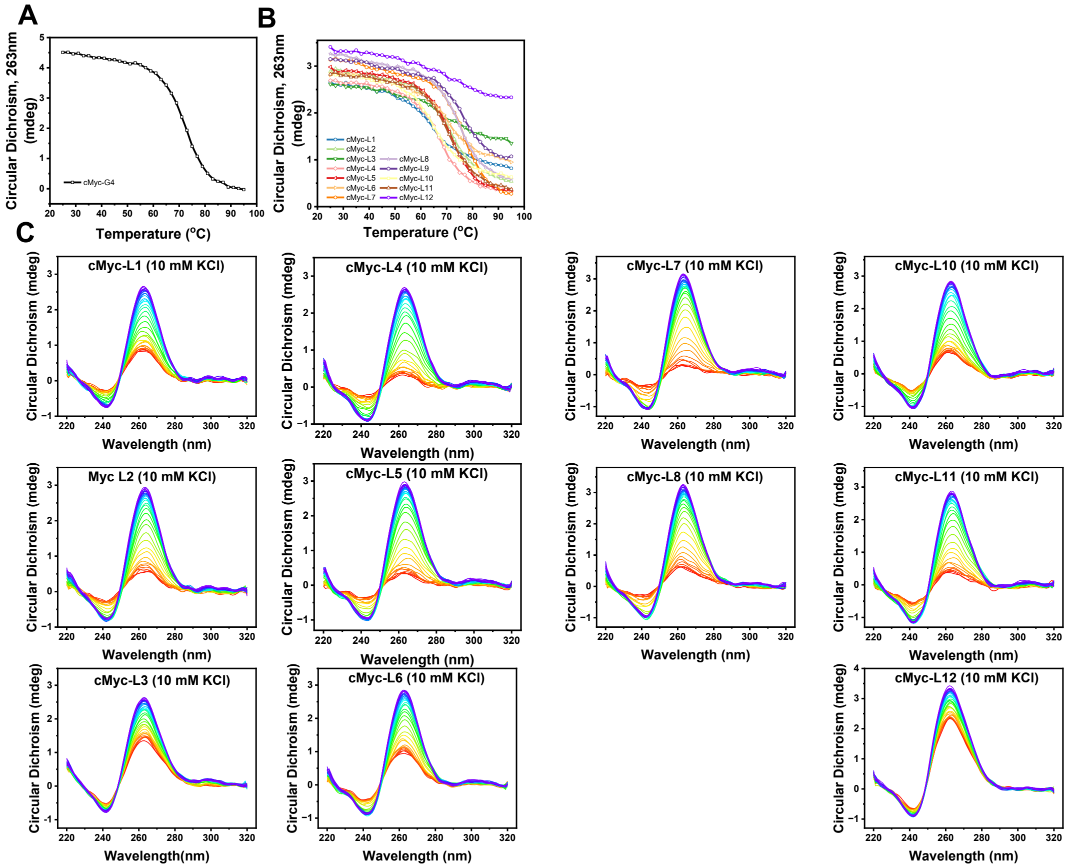

Figure S2. CD-melting spectra of cMyc-G4 and loop mutants. (A-B) Melting curves of cMyc-G4 and loop mutants in 10 mM KCl. **(C)** CD melting spectra of mutants in 10 mM KCl. Measurements were performed with 4 μM DNA sequences.

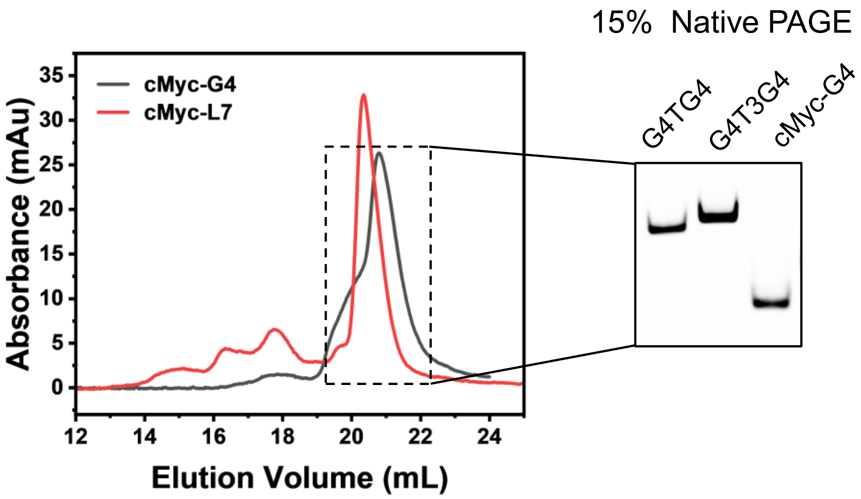

Figure S3. Assembly state of cMyc-G4 and loop mutant L7. The elution volumes of cMyc-G4 and L7 suggest that L7 maintains an intramolecular G4 structure with a slightly larger molecular contour than wild-type cMyc-G4. Experiments were conducted with 4 μM annealed DNA at 16°C in 100 mM KCl, with absorbance recorded at 260 nm. Native PAGE confirms cMyc-G4 as an intramolecular G4 structure. The first two bands represent tandem cMyc-G4 sequences connected by T or TTT linkers. Intermolecular G4 structures would migrate near these bands.

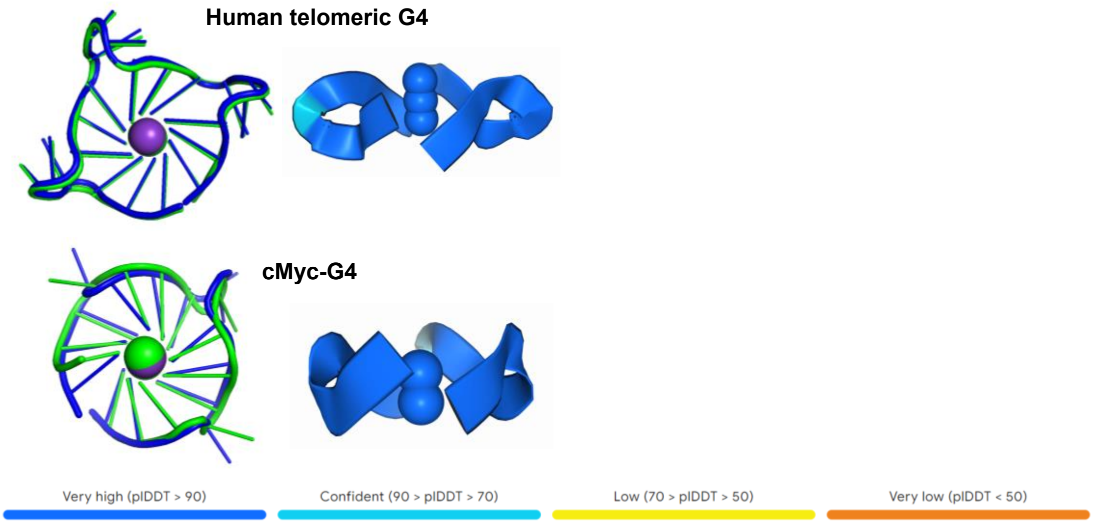

Figure S4. Alignment of AlphaFold 3-predicted and PDB-determined G4 structures. The alignment compares AlphaFold 3-predicted structures (blue) with PDB-determined structures (green). The right panel shows AlphaFold 3 confidence scores. For comparison, terminal nucleotides were trimmed from the sequences in PDB: one from human telomeric G4 (AGGGTTAGGGTTAGGGTTAGGG, 1kf1) and six from cMyc-G4 (1xav, TGAGGGTGGGTAGGGTGGGTAA). Minor inconsistencies in cMyc-G4 alignments may arise from adjacent nucleotides.

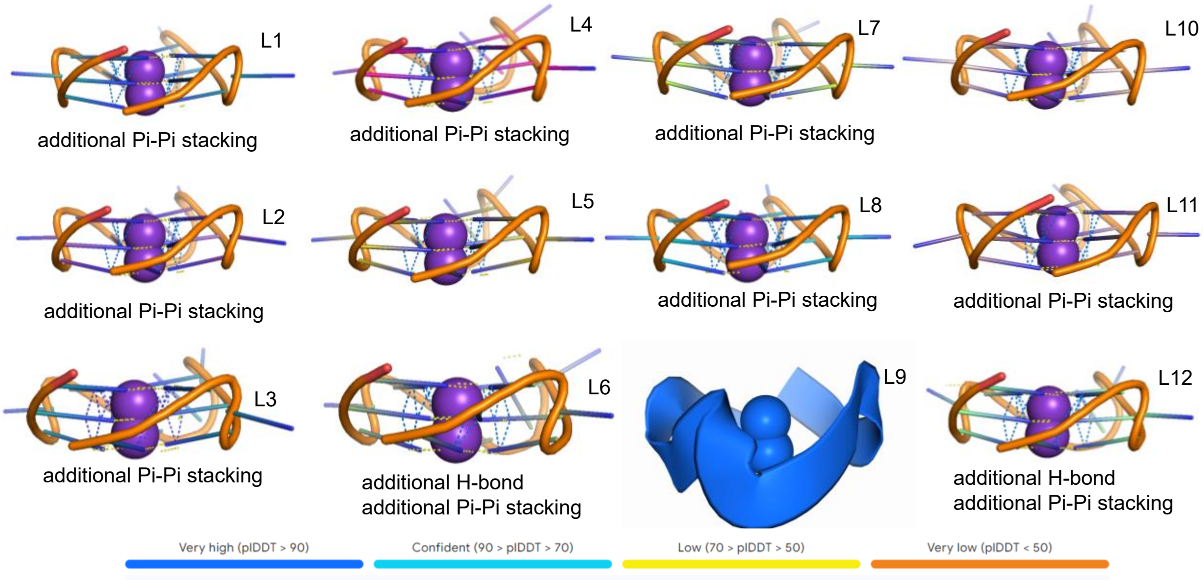

Figure S5. Predicted mutant G4 structures by AlphaFold 3. H-bonds (yellow) and π-π stacking (blue) in the mutant G4 structures reveal additional interactions compared to wild-type cMyc-G4 (Figure 2E). The L9 mutant demonstrates high prediction confidence, with structural details highlighted in Figure 2E.

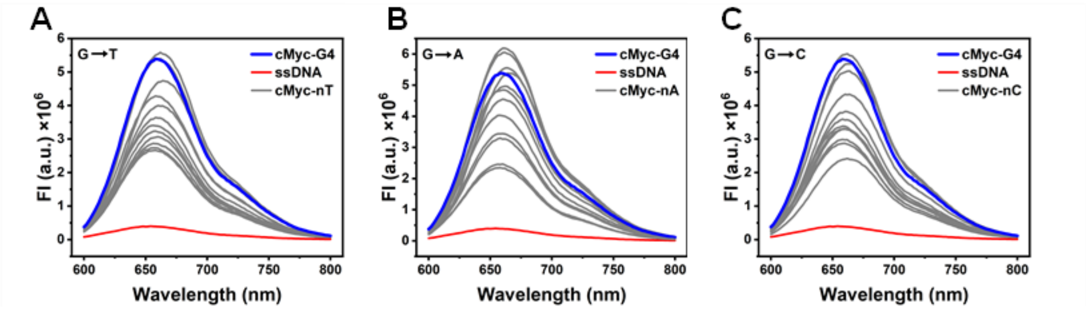

Figure S6. Fluorescence spectra of ISCH-1 probe with cMyc-G4 and G-run mutants. (A-C) Fluorescence spectra of ISCH-1 with cMyc-G4 (blue) and mutants (gray). A random-sequence ssDNA serves as a negative control (red). All experiments were performed with 1 µM annealed DNA at 25°C in 100 mM KCl.

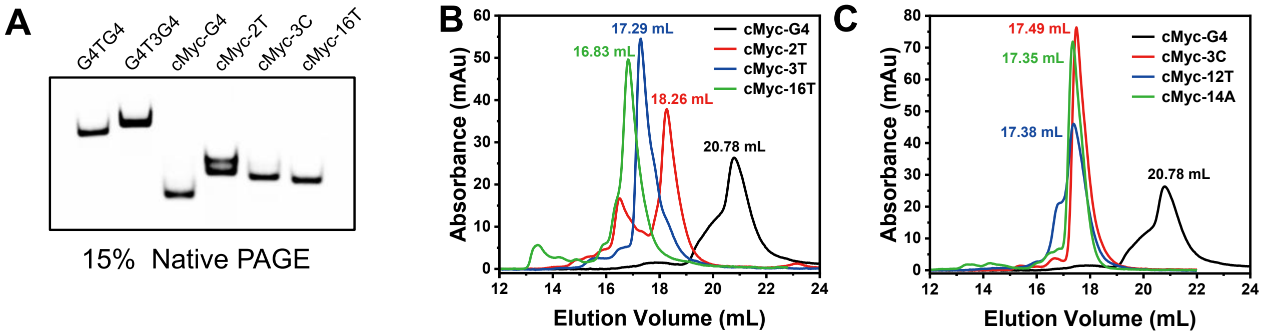

Figure S7. Assembly state of cMyc-G4 and G-run mutants. (A) Native PAGE analysis of 100 nM DNA samples at 16°C in 100 mM KCl. The first two bands represent tandem cMyc-G4 sequences connected by T or TTT linkers. Intramolecular G4 structures migrate similarly to wild-type cMyc-G4, as observed for 2T, 3C, and 16T. **(B-C)** Gel filtration chromatography of 4 µM DNA at 16°C in 100 mM KCl. The left shift in mutant elution profiles reflects looser structural organization compared to compact wild-type cMyc-G4. The two peaks for 2T correspond to its two folding conformations observed in native PAGE.

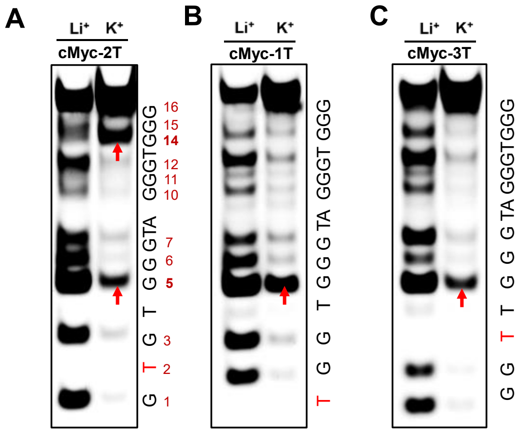

Figure S8. DMS-footprinting of G-run mutants 1T, 2T, and 3T. (A) DMS-footprinting of 2T in 100 mM LiCl and 100 mM KCl. Red arrows highlight significant cleavage at the 5th and 14th G in KCl, indicating their absence from Hoogsteen H-bond formation. **(B-C)** DMS-footprinting of 1T and 3T under the same conditions. The 5th G in both mutants is significantly cleaved in KCl, confirming it does not participate in Hoogsteen H-bonding.

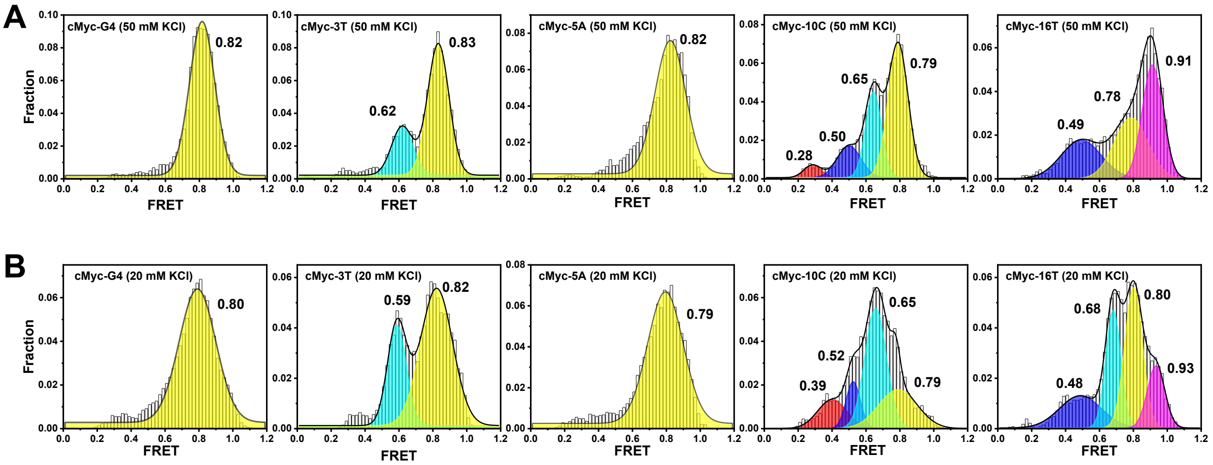

Figure S9. FRET histograms of cMyc-G4 and selected mutants. (A-B) FRET histograms of cMyc-G4, 3T, 5A, 10C, and 16T in 25 mM Tris-HCl buffer containing 50 mM and 20 mM KCl, respectively. Each histogram includes at least 300 traces.

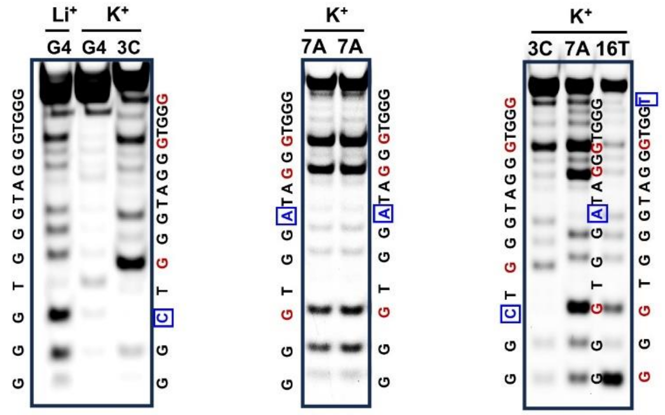

Figure S10. Independent replicate of DMS-footprinting analysis. This figure provides a biological replicate of the DMS-footprinting data shown in Figure 5B. The consistent cleavage patterns validate the proposed structural models. Mutated bases are highlighted in blue; guanines with prominent cleavage are marked in red.

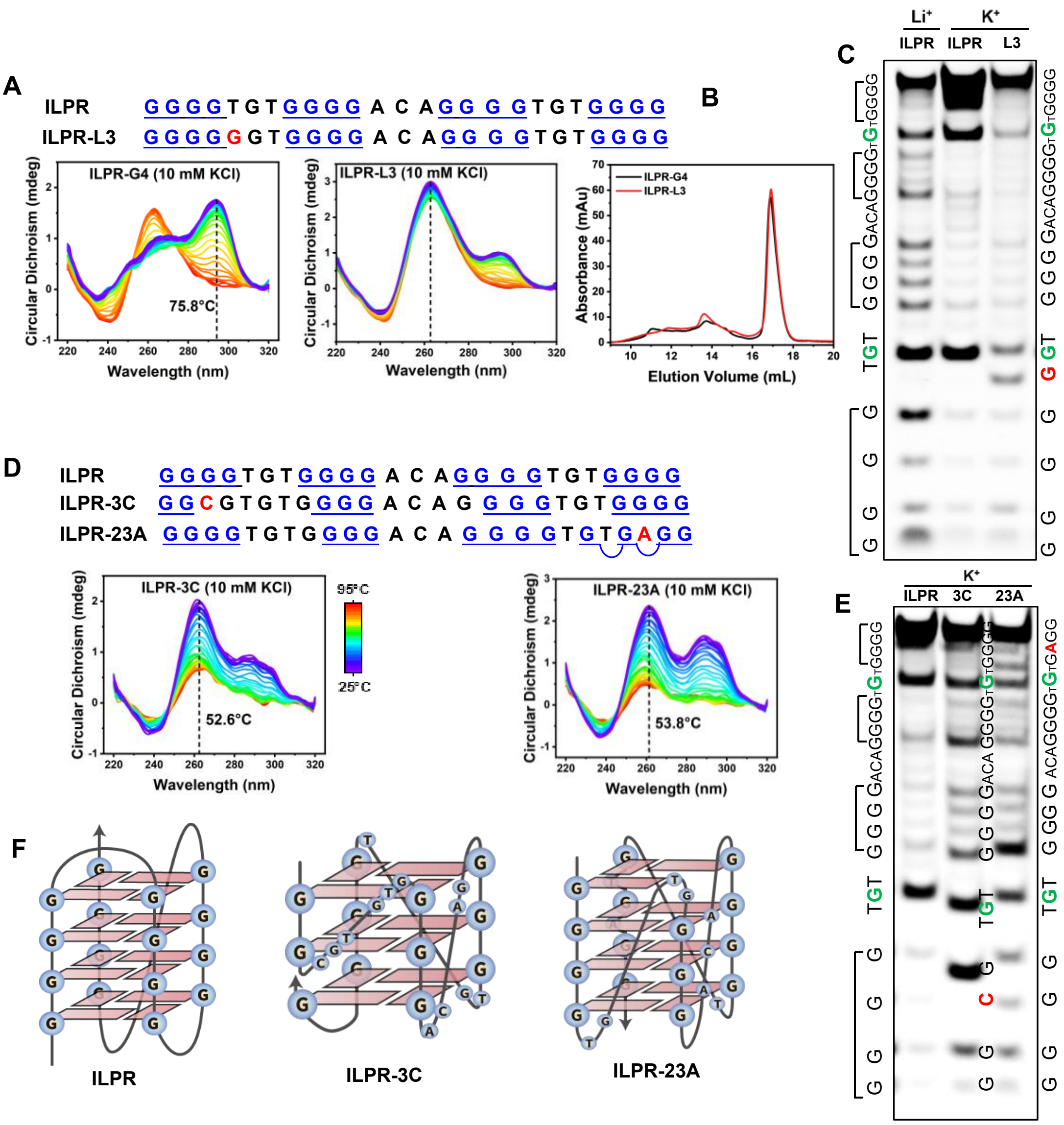

Figure S11. Structural consequences of loop and G-run mutations in the ILPR G4. (A) CD thermal melting profiles of ILPR G4 and the loop mutant L3 in 10 mM KCl. **(B)** gel-filtration chromatogram showing the same oligomeric state. **(C)** DMS-footprinting in 100 mM KCl. **(D)** CD thermal melting profiles of G-run mutants ILPR-3C and ILPR-23A in 10 mM KCl with melting temperatures indicated. **(E)** DMS-footprinting in 100 mM KCl. **(F)** Schematic structural models derived from the footprinting and CD data.

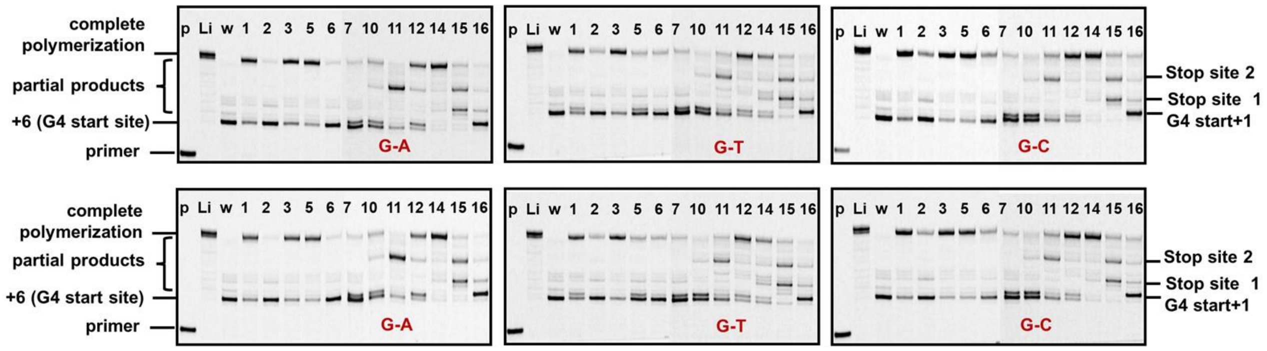

Figure S12. Representative gel images from independent replicates of the polymerase stop assays shown in Figure 6B. Lane designations are consistent with Figure 6: p, primer; Li, LiCl control; w, wild type in KCl; numbered lanes indicate G-run mutants.
